# Loss of Anti-Bax Function in Gerstmann-Sträussler-Scheinker Syndrome-Associated Prion Protein Mutants

**DOI:** 10.1371/journal.pone.0006647

**Published:** 2009-08-14

**Authors:** Julie Jodoin, Micheal Misiewicz, Priya Makhijani, Paresa N. Giannopoulos, Jennifer Hammond, Cynthia G. Goodyer, Andréa C. LeBlanc

**Affiliations:** 1 Bloomfield Center for Research in Aging, Lady Davis Institute for Medical Research, Sir Mortimer B. Davis Jewish General Hospital, Montréal, Canada; 2 Department of Neurology and Neurosurgery, McGill University, Montréal, Canada; 3 Department of Pediatrics, McGill University, Montréal, Canada; University of Oulu, Finland

## Abstract

Previously, we have shown the loss of anti-Bax function in Creutzfeldt Jakob disease (CJD)-associated prion protein (PrP) mutants that are unable to generate cytosolic PrP (CyPrP). To determine if the anti-Bax function of PrP modulates the manifestation of prion diseases, we further investigated the anti-Bax function of eight familial Gerstmann-Sträussler-Scheinker Syndrome (GSS)-associated PrP mutants. These PrP mutants contained their respective methionine (^M^) or valine (^V^) at codon 129. All of the mutants lost their ability to prevent Bax-mediated chromatin condensation or DNA fragmentation in primary human neurons. In the breast carcinoma MCF-7 cells, the F198S^V^, D202N^V^, P102L^V^ and Q217R^V^ retained, whereas the P102L^M^, P105L^V^, Y145stop^M^ and Q212P^M^ PrP mutants lost their ability to inhibit Bax-mediated condensed chromatin. The inhibition of Bax-mediated condensed chromatin depended on the ability of the mutants to generate cytosolic PrP. However, except for the P102L^V^, none of the mutants significantly inhibited Bax-mediated caspase activation. These results show that the cytosolic PrP generated from the GSS mutants is not as efficient as wild type PrP in inhibiting Bax-mediated cell death. Furthermore, these results indicate that the anti-Bax function is also disrupted in GSS-associated PrP mutants and is not associated with the difference between CJD and GSS.

## Introduction

Autosomal dominant inherited prion protein (PrP) gene mutations cause approximately 10% of all prion diseases [1]. Eleven different single point mutations are associated with Creutzfeldt-Jakob disease (CJD), eight different single point mutations are associated with Gerstmann-Straüssler-Scheinker (GSS), and the D178N mutation causes Fatal Familial Insomnia (FFI) [2]–[4]. Each mutation is specifically associated with either a methionine or a valine polymorphic amino acid at codon 129, which can severely affect the type, the onset and the duration of the disease [4]. While each of the PrP single point mutations is sufficient for neurodegeneration, the onset and duration of disease, the area of the brain affected and the pathology associated with neurodegeneration distinguish each of these diseases. CJD, characterized by spongiform encephalopathy, astrogliosis, and proteinase K resistant PrP in the cerebral cortex, generates a rapidly progressing dementia in affected individuals [3], [4]. FFI affects the ventral thalamic area and the primary symptoms are insomnia followed by a rapidly progressing dementia of short duration [5]. GSS, characterized by astrogliosis, proteinase K resistant PrP, PrP-plaques, and sometimes spongiform encephalopathy and neurofibrillary tangles, is associated with ataxia and/or spastic paraparesis [2], [6]. GSS occurs in the fifth or sixth decade of life and, in contrast to CJD, the progression of the disease is slow [1].

The underlying molecular mechanism of prion diseases associated with each of these mutations is unclear. Gain of function is suspected as the cause of the disease. However, while the mutant proteins are expressed throughout the lifespan of the individual, disease rarely develops before the fourth or fifth decade of life, indicating that misfolding of PrP caused by the mutation is not sufficient to initiate the neurodegeneration. This prompted us to investigate if loss of function could be involved in the length and severity of disease progression.

While the normal function of PrP is still not entirely clear, there is growing evidence for the neuroprotective roles of PrP [7], [8]. PrP null hippocampal neuronal cell lines and PrP null mice have increased susceptibility to a variety of insults [9]–[13]. Furthermore, recent evidence indicates that PrP prevents cell death in cancer cell types. PrP prevents apoptosis in multidrug-resistant breast and gastric carcinoma cells [14]–[17]. We have reported that the normal cellular prion protein prevents Bax-mediated cell death in primary human neurons and in the breast carcinoma MCF-7 cell line [18]–[20]. Bax, a member of the Bcl-2 family of proteins, is a strong pro-apoptotic protein in neurons [21]. Bax is normally cytosolic or loosely attached to mitochondria but, in cells submitted to apoptotic insults, Bax undergoes a change in conformation, oligomerisation, translocation to the mitochondria and then induces the release of apoptogenic factors from the mitochondria into the cytosol [22]. The release of cytochrome c in the cytosol activates the apoptosome, resulting in Caspase-9 (Casp9) and Caspase-3 (Casp3) activation and caspase-dependent DNA fragmentation into 180 bp fragments and condensed chromatin [23]. The mitochondria-released apoptosis inducing factor (AIF) and endonuclease G (Endo G) directly translocate to the nucleus and activate caspase-independent DNA fragmentation in large 50 Kb fragments and condensed chromatin [24], [25]. PrP prevents the conformational change of Bax and thus acts at the very first step of Bax activation [18]. While most of the PrP accumulates on the cell surface as a GPI-anchored protein, it is the smaller amount of cytosolic PrP (CyPrP) that is responsible for the anti-Bax function [19], [26]. CyPrP can arise from either retrotranslocation of PrP from the secretory pathway or via a failure of PrP's signal peptide to integrate into the endoplasmic reticulum during protein synthesis (pre-emptive quality control pathway) [19], [27]–[29]. Moreover, the anti-Bax function of PrP is situated within helix 3 of PrP, the area with the most single point mutations in PrP diseases [30].

We previously showed that the GSS-associated A117V PrP mutant with a valine at codon 129 retained the anti-Bax function while all of the 11 CJD-associated PrP mutations carrying a methionine at codon 129 or the FFI-associated D178N PrP mutant lost the ability to prevent Bax-mediated condensed chromatin in human neurons [31]. Considering that GSS disease has a slower progression and a later onset compared to CJD, we investigated whether all other GSS-associated PrP mutants retained the anti-Bax function.

## Materials and Methods

### Cell cultures

Cultures of human primary neurons were done as previously described under the ethical approval from the McGill University Institutional Review Board [32]. Mouse neuroblastoma N2a cells and human MCF-7 breast carcinoma cells were obtained from The American Type Culture Collection (Manassas, VA, USA) and cultured as described previously [30].

### Cloning, site-directed mutagenesis of human PrP, and sequencing of PrP mutants

GSS-associated mutations were produced by QuikChange Site-Directed Mutagenesis (Invitrogen, Burlington, ON, Canada) from the pcDNA3.1(+)-PrP wild type carrying a valine (WT PrP^V^) or a methionine (WT PrP^M^) at the polymorphic codon 129 [31]. Forward primers used to produce PrP mutants P102L^M^, P102L^V^, P105L^V^, Y145stop^M^, F198S^V^, D202N^V^, Q212P^M^, and Q217R^V^ are the following: F198S, 5′-AGGGGGAGAACTCCACCGAGACCGAC-3′; D202N, 5′-GAACTTCACCGAGACCAACGTTAAGATGATGGAG-3′; Q217R, 5′-CAGATGTGTAT CACCCGGTACGAGAGGGAATCTC-3′; P102L, 5′-GTCAGTGGAACAAGCTGAGTAAGC CAAAAACC-3′; P105L, 5′-GGAACAAGCCGAGTAAGCTAAAAACCAACATGAAGCAC-3′; Y145stop, 5′-CGGCAGTGACTAGGAGGATCGTTACTATCG-3′; and Q212P, GCGTGGTTGAGCC GATGTGTATCACCC. All PrP mutations were confirmed by sequencing using the Sequenase Version 2.0 DNA Sequencing kit (USB, Cleveland, OH, USA) and the polymorphism at codon 129 was confirmed by PCR [31]. As previously done for WT PrP, PrP mutant cDNAs were PCR amplified and cloned downstream of the cytomegalovirus (CMV) promoter of the pBudCE4.1 bigenic vector containing the enhanced green fluorescent protein (EGFP) or EGFP-Bax cDNAs under the elongation factor 1α (EF-1α) promoter [18]. The PCR amplified PrP mutants were cloned into the *Sca* I and *Xba* I sites of pBud-EGFP or pBud-EGFP-Bax generating pBud-EGFP/PrP mutants or pBud-EGFP-Bax/PrP mutants.

### Transfections

Human neurons (1.5×10^5^ cells) plated onto poly-L-lysine-coated plastic coverslips and MCF-7 cells (2.5×10^5^ cells) plated onto glass coverslips were transfected with the Helios Gene Gun system (Bio-Rad, Mississauga, ON, Canada) at a shooting pressure of 100 psi for human neurons and 220 psi for MCF-7 cells. Cartridges contained 1 µg DNA and 0.125 mg gold per shot [18], [31]. N2a cells (0.8×10^6^ cells) plated in six-well plates were transfected with 4 µg of pBud-EGFP/PrP or/PrP mutant DNAs using the Lipofectamine^2000^ reagent (Invitrogen). To generate SP-CyPrP, N2a cells were transfected with 10 and 20 µg of pBud-EGFP/PrP DNA, as previously described [27].

### Condensed chromatin

Twenty hours after transfection, MCF-7 cells and human neurons were washed with phosphate-buffered saline (PBS, 150 mM NaCl, 2.7 mM KCl, 1.3 mM KH_2_PO_4_, and 8.1 mM Na_2_HPO_4_, pH 7.4), fixed 20 min at room temperature in 4% paraformaldehyde and 4% sucrose in PBS, and the chromatin stained 20 min with 1 µg/mL Hoechst 33342 (Sigma-Aldrich, Oakville, ON, Canada) in PBS. The percentage of cell death was calculated by counting the number of EGFP-positive cells with condensed chromatin over the total number of EGFP-positive cells using a Nikon Eclipse TE2000-U microscope.

### TUNEL

Human neurons were fixed 20 hours after transfection, as described above, and Terminal deoxynucleotidyl transferase-mediated dUTP nick end labeling (TUNEL) was performed with the TM Red *In Situ* Cell Death Detection kit (Roche Applied Science, Laval, QC, Canada). The percentage of TUNEL-positive cells was obtained by counting the TUNEL- and EGFP-positive cells over the total number of EGFP-positive cells.

### Caspase activity

MCF-7 cells were labeled 20 hours after transfection for active caspases with the SR Fluorochrome-labeled inhibitors of caspases (FLICA) poly caspase kit (AbD Serotec, Raleigh, NC, USA). The percentage of FLICA-positive cells was calculated by counting the FLICA- and EGFP-positive cells over the number of total EGFP-positive cells.

### Subcellular Fractionations

N2a cells were treated 24 hours after transfection with 0.25 µM epoxomycin (BioMol, Plymouth Meeting, PA, USA) and 5 µg/mL Brefeldin A (BioMol) for 18 hours. Subcellular fractionation was performed as described previously [31] using one well of a six-well plate for each condition. To prepare samples for western blotting, proteins from the homogenate (1/20 of one well), membranes (1/4 of one well), cytosol (1 well), and nuclei (1/4 of one well) were precipitated with four volumes of ice-cold methanol overnight at −20°C, centrifuged at 11 500×*g* 15 min at 4°C, dried, and solubilized in the Laemmli's sample buffer (2% SDS (w/v), 5% β-mercaptoethanol (v/v), 10% glycerol, 0.01% bromophenol blue (w/v), and 62.5 mM Tris-HCl, pH 6.8).

### Detergent solubility of CyPrP

Cytosolic proteins obtained from the subcellular fractions were solubilized 20 min on ice in lysis buffer (150 mM NaCl, 2 mM EDTA, 0.5% Triton X-100 (v/v), 0.5% sodium deoxycholate (NaDOC) (w/v), 50 mM Tris-HCl, pH 7.5) and centrifuged at 11 500×*g* for 15 min at 4°C to separate detergent-soluble proteins (supernatant) from the detergent-insoluble proteins (pellet). Detergent-soluble proteins were concentrated by methanol precipitation and these as well as the detergent insoluble proteins solubilized in the Laemmli's sample buffer for western blotting.

### Peptide-N-glycosidase F digestion

Membrane fractions from N2a cells were treated with Peptide-N-glycosidase F (PNGase F) according to manufacturer's instructions (New England BioLabs, Pickering, ON, Canada). Briefly, proteins from membrane fractions were adjusted to 0.5% SDS and 1% β-mercaptoethanol, denatured 10 min at 100°C and incubated overnight at 37°C with 2 units of PNGase F in the presence of protease inhibitors. The proteins were methanol precipitated as described above.

### Western blot analyses

The proteins were separated on 15% SDS-PAGE gels and transferred to polyvinylidene fluoride membranes. The membranes were probed with the anti-PrP 3F4 antibody [33] (1∶2000), the polyclonal antiserum directed against the N-terminal signal peptide of PrP (SP-PrP, 1∶1000, kindly provided by Dr. D.A. Harris, Washington University School of Medicine), the mitochondrial heat shock protein 70 (mtHsp70, 1∶10 000, clone JG1; Affinity BioReagents, Golden, CO, USA), CHOP (also called GADD153, 1∶1000, clone B-3; Santa Cruz Biotechnology, Santa Cruz, CA, USA), Poly-(ADP-ribose)-polymerase (PARP, 1∶2000; Roche Applied Science), Bax (1∶1000, Clone N20; Santa Cruz Biotechnology), BiP (also called GRP 78, 1∶250, clone H-129; Santa Cruz Biotechnology), GFP (1∶1000, clone B-2; Santa Cruz Biotechnology), and β-actin (1∶1000, clone AC-15; Sigma-Aldrich). Immunoreactivity was detected with anti-mouse or anti-rabbit secondary antibodies conjugated to horseradish peroxidase (GE Healthcare, Baie d'Urfe, QC, Canada) and chemiluminescence reagents (Millipore, Billerica, MA, USA).

### Quantitation of cytosolic and nuclear PrP

The intensity of PrP detected by western blot analyses was resolved with the Image Quant TL software (GE Healthcare). The ratio of cytosolic PrP over membrane PrP or the ratio of nuclear PrP over membrane PrP was calculated.

### Statistical analyses

Statistical analyses were performed with StatView software (SAS Institute, Cary, NC, USA). To determine the statistically significant difference, we used ANOVA followed by a *post hoc* Scheffé test. A *p* value≤0.05 was considered a significant difference. To determine the correlation coefficient and its significance, we used the Fisher's *r* to *z* transformation.

## Results

### GSS-associated PrP mutants F198S^V^, D202N^V^, P102L^V^ and Q217R^V^, but not P102L^M^, P105L^V^, Y145stop^M,^ and Q212P^M^, prevent Bax-mediated condensed chromatin in MCF-7 cells

**Figure 1:**
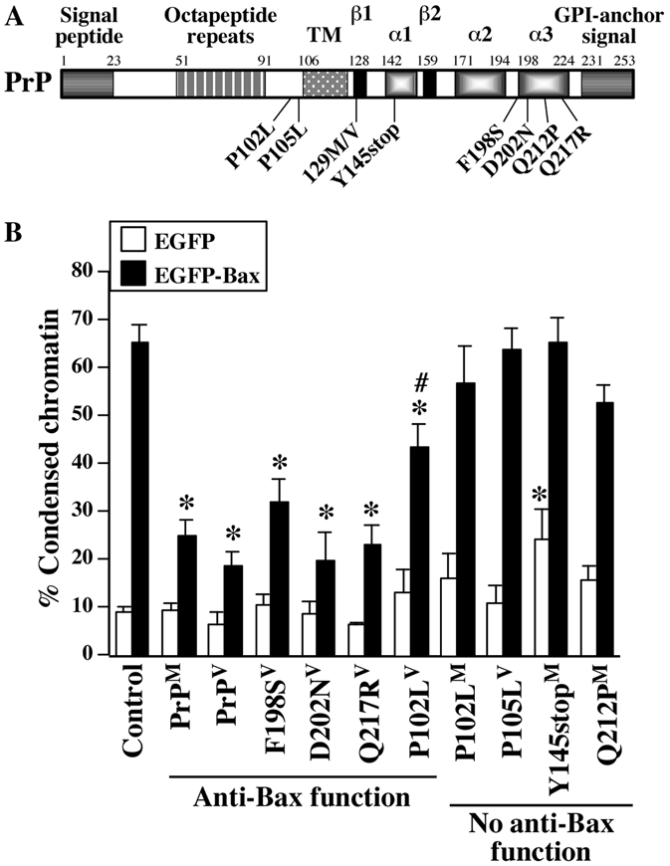
GSS-associated PrP mutants F198S^V^, D202N^V^, P102L^V^ and Q217R^V^, but not P102L^M^, P105L^V^, Y145stop^M,^ and Q212P^M^, prevent Bax-mediated condensed chromatin in MCF-7 cells. A. Schematic diagram of human PrP showing the signal peptide, the octapeptide repeats, the transmembrane domain (TM), the β-sheets (β1 and β2), the α-helices (α1, α2, α3), the glycosyl phosphatidylinositol (GPI)-anchor signal peptide, the polymorphic codon at amino acid 129, and the GSS PrP mutations. B. Percentage of cell death assessed by condensed chromatin in MCF-7 cells transfected with pBud-EGFP or pBud-EGFP-Bax (control), pBud-EGFP/PrP or PrP mutants, and pBud-EGFP-Bax/PrP or PrP mutants carrying a methionine (^M^) or a valine (^V^) at codon 129. Data represent the mean±SEM of six independent experiments. At least 600 cells were counted for each condition. * and # indicate a statistically significant difference (*p*≤0.05) compared to the control and wild type PrP, respectively.

GSS-associated PrP mutations P105L, A117V, F198S, D202N, and Q217R have been reported with a valine at codon 129, whereas P102L, Y145stop, and Q212P carry a methionine at codon 129 [1], [3], [34]–[36]. In some rare cases, P102L is associated with a valine at codon 129 [34]. To assess if the PrP anti-Bax function was altered in GSS mutants, specific single point mutations with their respective methionine or valine at codon 129 into the prion protein cDNA were produced and the mutant cDNA inserted into the bigenic pBudCE4.1 construct under the CMV promoter (**Fig. 1A**). This construct also expresses either EGFP or EGFP-Bax protein under the EF-1α promoter. Therefore, cell death is specifically induced by over-expression of Bax and the anti-Bax function of co-expressed wild type (WT) PrP or PrP mutants is directly assessed. Bax overexpression resulted in the condensation of chromatin in approximately 65% of the transfected MCF-7 cells within 20 hrs of transfection (**Fig. 1B**). Co-expression of WT PrP with either a methionine (PrP^M^) or valine (PrP^V^) at codon 129 prevented Bax-mediated condensed chromatin. Similar to our previous observations with CJD mutants, the P102L^M^, P105L^V^, Y145stop^M^ and Q212P^M^ PrP mutants completely lost their ability to prevent Bax-mediated condensed chromatin. However, F198S^V^, D202N^V^, and Q217R^V^ completely while the P102L^V^ partially retained the anti-Bax function. None of the GSS-associated PrP mutants co-expressed with EGFP induced chromatin condensation in MCF-7 cells, except for the Y145stop^M^ which, consistent with previous observations [37], significantly increased the number of cells with condensed chromatin by 15% (**Fig. 1B**). Together, these results indicate that only a few of the GSS-associated mutants retain PrP's ability to prevent Bax-mediated condensed chromatin in MCF-7 cells.

### GSS-associated PrP mutants F198S^V^, D202N^V^, and Q217R^V^ cannot inhibit Bax-mediated caspase activation

**Figure 2:**
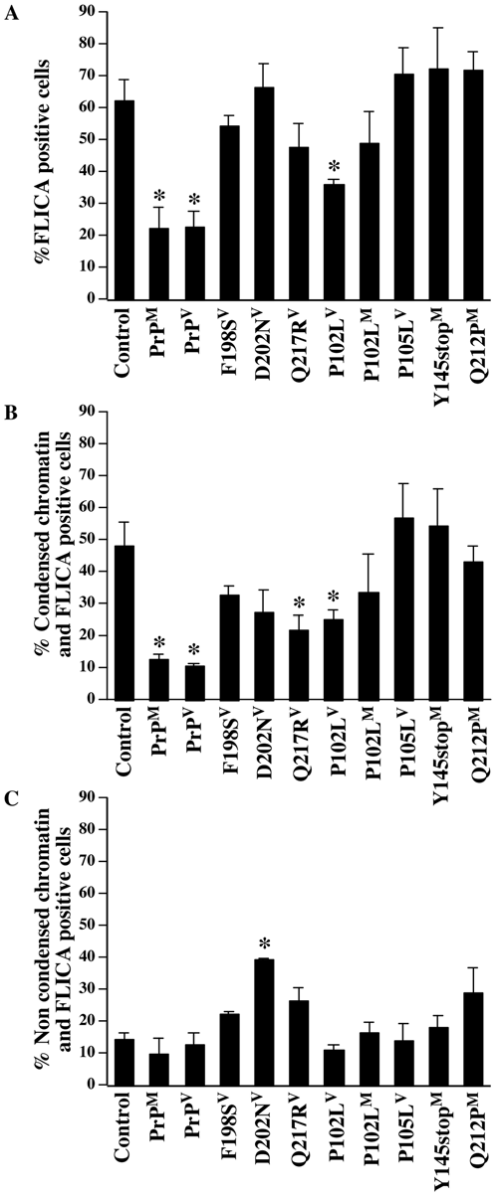
GSS-associated PrP mutants F198S^V^, D202N^V^, and Q217R^V^ cannot inhibit Bax-mediated caspase activation. A. Percentage of FLICA positive MCF-7 cells transfected with pBud-EGFP-Bax (control) or pBud-EGFP-Bax/PrP or PrP mutants. B & C. Percentage of FLICA positive and condensed chromatin (B) or FLICA positive and non-condensed chromatin (C) transfected MCF-7 cells. For A–C, data represent the mean±SEM of three independent experiments. At least 300 cells were counted for each condition. * indicates a statistically significant difference (*p*≤0.05) compared to the control.

MCF-7 cells are deficient in Caspase-3 (Casp3) expression because of a deletion in the Casp3 gene and thus cannot undergo DNA fragmentation in apoptotic conditions [38], [39]. Condensed chromatin is dependent on the activation of Casp6 and Casp7 in these cells. To determine if the F198S^V^, D202N^V^, and Q217R^V^ PrP mutants completely inhibited Bax-mediated apoptosis or simply delayed condensed chromatin formation, we measured caspase activity with the FLICA pan-caspase substrate, SR-VAD-fmk (**Fig. 2A**). Bax induced caspase activity in approximately 60% of the cells and WT PrP^M^ and PrP^V^ inhibited caspase activation. However, in contrast to cell death measured by condensed chromatin, only P102L^V^ inhibited caspases activated by EGFP-Bax overexpression. FLICA positive cells did not always contain condensed chromatin (**Fig. 2B**). In condensed chromatin-positive cells, Q217R^V^ and P102L^V^, but not the F198S^V^ or D202N^V^, inhibited Bax-mediated caspase activation. Furthermore, D202N^V^ significantly induced caspase activation in non-condensed chromatin cells (**Fig. 2C**). These results indicate that the GSS-associated PrP mutants, F198S^V^, D202N^V^, and Q217R^V^, simply delay the condensation of chromatin but do not completely inhibit Bax-mediated apoptotic pathways in MCF-7 cells.

### Decreased levels of CyPrP correlate with the loss of anti-Bax function in GSS-associated PrP mutants expressed in MCF-7 cells

**Figure 3:**
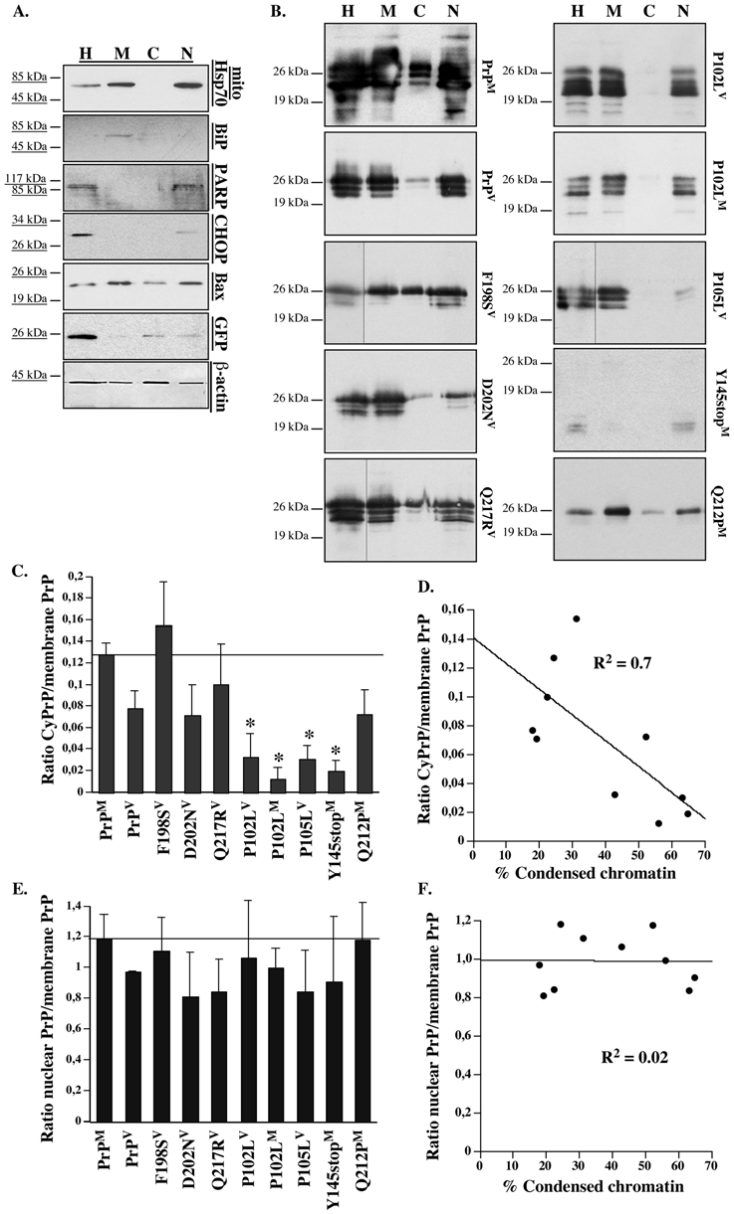
Correlation of the anti-Bax activity with CyPrP levels in GSS-associated PrP mutants. A. Western blot with mitochondrial Hsp70 (mtHsp70), Bip, PARP, CHOP, Bax, GFP, and β-actin in protein extracts from subcellular fractions from pBud-EGFP/PrP^M^-transfected N2a cells. Homogenates (H), membrane (M), cytosol (C), and nuclear (N) protein extracts were loaded on gels as a ratio of 1∶5∶20∶5, respectively. B. Western blots of PrP with the 3F4 antibody in proteins extracted from subcellular fractions isolated from pBud-EGFP/PrP or PrP mutant-transfected N2a cells. C. Ratio of CyPrP to membrane PrP. The data represent the mean±SEM of three to four independent experiments. * indicates a statistically significant difference (*p*≤0.05) compared to WT PrP. D. Correlation between the levels of CyPrP and levels of condensed chromatin. The correlation is statistically significant (*p*≤0.05) as determined by a Fisher's *r* to *z* transformation. E. Ratio of nuclear PrP to membrane PrP. The data represent the mean±SEM of three to four independent experiments. F. Correlation between the levels of nuclear PrP and levels of condensed chromatin. The correlation is not statistically significant (*p* = 0.96).

To determine if the loss of anti-Bax function from the GSS-associated PrP mutants is due to lower levels of CyPrP as previously shown in CJD mutants [31], transfected cells treated with Brefeldin A to promote retrotranslocation and epoxomicin to prevent proteasomal degradation of CyPrP, were submitted to subcellular fractionation (**Fig. 3A**). Mouse N2a cells were used for subcellular fractionations because Brefeldin A and epoxomicin treatment of MCF-7 cells increase the expression of endogenous human PrP and the 3F4 antibody can be used to selectively recognize the human PrP expressed from the transfected construct [31]. The proteins extracted from the membrane, cytosolic, and nuclear fractions showed the presence of mitochondrial Hsp70 (mtHsp70) in the membrane and nuclear fraction only, the presence of the endoplasmic reticulum (ER)-localized BiP protein in the membranes only, and the presence of nuclear protein PARP and nuclear-translocated protein CHOP [40] in the nuclear fraction. Bax was detected in all fractions and EGFP was present in the nuclear and cytosolic, but not in the membrane fractions. The presence of mtHsp70 and Bax in the nuclear fraction, in addition to the nuclear markers PARP and CHOP, indicates either a contamination of the nuclear proteins or a redistribution of these proteins into the nucleus under Brefeldin A and epoxomicin treatment. Nevertheless, these results indicated that the cytosolic and membrane fractions are pure and can thus be used to assess the presence of CyPrP.

The WT PrP^M^, WT PrP^V^, F198S^V^, D202N^V^, Q217R^V^ and Q212P^M^ PrP mutants generated detectable levels of CyPrP while the P102L^M^, P102L^V^, P105L^V^, and Y145stop^M^ did not (**Fig. 3B**). The levels of PrP in the membrane, cytosolic and nuclear subcellular fractions were then analyzed quantitatively and the levels of CyPrP were expressed relative to the membrane PrP to account for differences in the levels of protein expression (**Fig. 3C**). Except for the Q212P^M^, the mutants generating the highest levels of CyPrP were those inhibiting Bax-mediated condensed chromatin, whereas those with the lowest levels of CyPrP were the ones that had partially or completely lost this function. The level of CyPrP correlated significantly with their ability to prevent Bax-mediated condensed chromatin (**Fig. 3D**). In contrast, the ratio of nuclear PrP relative to membrane PrP was similar in WT and mutant PrPs (**Fig. 3E**) and did not correlate with the level of condensed chromatin (**Fig. 3F**). The results show that reduced CyPrP correlates with loss of PrP's anti-Bax function in MCF-7 cells.

### Glycosylation profile of GSS-associated mutants does not correlate with anti-Bax activity

**Figure 4:**
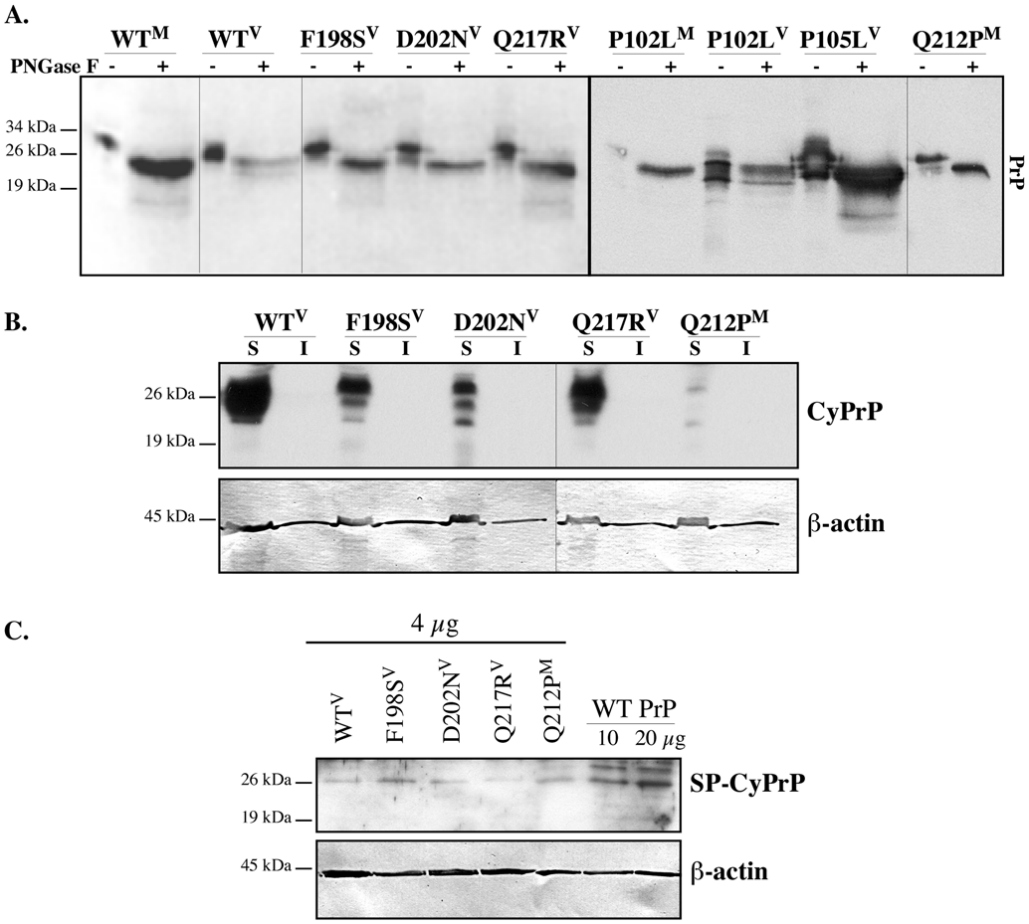
Characterization of the glycosylation profile, detergent solubility and type of CyPrP in GSS mutant-transfected cells. A. Western blots of PrP in membrane fractions from pBud-EGFP/PrP or PrP mutant-transfected cells treated with or without PNGase F. B. Western blot with 3F4 anti-PrP or anti-β-actin showing detergent solubility of CyPrP from pBud-EGFP/PrP^V^ or PrP mutant-transfected N2a cells. S indicates detergent soluble and I indicates detergent insoluble CyPrP. C. Western blots with anti-SP-CyPrP and β-actin in cytosolic proteins of N2a cells transfected with 4 µg of pBud-EGFP/PrP^V^ or PrP mutants or transfected with 10 µg and 20 µg of pBud-EGFP/PrP^V^ as positive control for SP-CyPrP detection.

Treatment with Brefeldin A prevents maturation of secretory proteins through the Golgi apparatus, therefore only unglycosylated or immature glycosylated forms of PrP were expected in the transfected cells. However, the GSS PrP mutants showed differences in their glycosylation profile (**Fig. 3B**). In total cellular homogenates or in the membrane fractions, WT PrP^M^ or^ V^, P102L^M^ or^ V^, P105L^V^, Q217R^V^ showed three PrP immunoreactive protein bands, while F198S^V^ and D202N^V^ generated two, and the Q212P^M^ showed only one. The Y145stop^M^ showed two lower MW protein bands that cannot represent glycosylation since the glycosylation sites are absent in this truncated mutant. To determine if these protein bands reflected glycosylation or truncations of the PrP mutants, the membrane proteins were deglycosylated with PNGase F (**Fig. 4**). Deglycosylation converted all mutant proteins to the 25 kDa deglycosylated PrP. In addition, the WT PrP^M^, F198S^V^, Q217R^V^, P102L^V^ and P105L^V^ displayed very low levels of some 14–23 kDa MW fragments indicating a small amount of truncated PrP. There was no correlation between the glycosylation profile and the anti-Bax function. These results indicate that the GSS-associated mutants are differentially glycosylated in these transfected cells but that the glycosylation profile does not account for the anti-Bax function of PrP.

### GSS mutant CyPrPs do not aggregate

To study if CyPrP mutants aggregate, cytosolic proteins from WT PrP or PrP mutant transfected cells were solubilized in 0.5% Triton X-100 and 0.5% sodium deoxycholate. WT PrP^V^ as well as PrP mutants F198S^V^, D202N^V^, Q217R^V^, and Q212P^M^ generated detergent soluble, but no detergent insoluble CyPrP (**Fig. 4B**). The results show that CyPrP generated from GSS PrP mutants does not aggregate in the cytosol and thus, aggregation cannot be responsible for the loss of anti-Bax function in the GSS mutants that generate CyPrP.

### Transfection of WT PrP and GSS PrP mutants generate some CyPrP through the pre-emptive quality control pathway

To determine if CyPrP generated from GSS PrP mutants occurs through the pre-emptive quality control pathway and thus has retained its signal peptide, we performed a western blot of the cytosolic proteins with an antibody directed against the N-terminal signal peptide of PrP (SP-PrP) [27]. CyPrP from WT PrP^V^, PrP F198S^V^, D202N^V^, Q217R^V^, and Q212P^M^ all contained a small amount of SP-CyPrP, whereas increasing the concentration of WT PrP cDNA to 10 or 20 µg generated easily detectable SP-CyPrP (**Fig. 4C**). Given that the CyPrP easily detected with the 3F4 antibody migrates at 25 kDa, the expected size for PrP that would have lost both its N-terminus and C-terminus signal peptides, these results indicate that GSS PrP mutants generate considerably more retrotranslocated CyPrP than SP-CyPrP.

### All GSS-associated PrP mutants lose their anti-Bax function in primary human neurons

**Figure 5:**
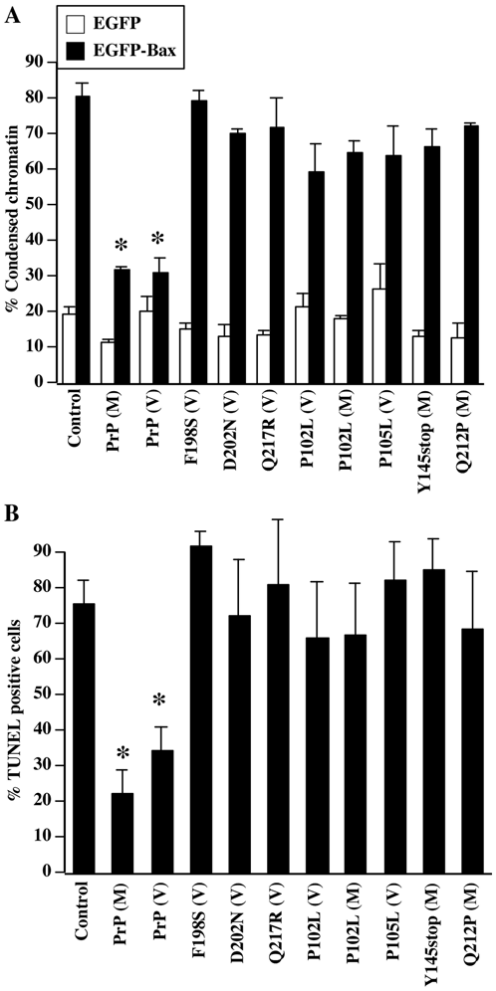
All GSS-associated PrP mutants lose their anti-Bax function in primary human neurons. A. Percentage of cell death assessed by condensed chromatin in human neurons transfected with pBud-EGFP or pBud-EGFP-Bax (control), pBud-EGFP/PrP or PrP mutants, and pBud-EGFP-Bax/PrP or PrP mutants carrying a methionine (^M^) or a valine (^V^) at codon 129. Data represent the mean±SEM of three independent experiments. At least 300 cells were counted for each condition. * indicates a statistically significant difference (*p*≤0.05) compared to the control. B. Percentage of apoptotic cells displaying DNA fragmentation assessed by TUNEL in human neurons transfected with pBud-EGFP-Bax (control), pBud-EGFP-Bax/PrP, or pBud-EGFP-Bax/PrP mutants. Data represent the mean±SEM of three independent experiments. At least 150 cells were counted for each condition. * indicates a statistically significant difference (*p*≤0.05) compared to the control.

To determine the anti-Bax function of GSS-associated PrP mutants in a neuronal cell type, we assessed the ability of the GSS-associated mutants against Bax-mediated cell death in primary human neurons. None of the GSS-associated PrP mutants induced cell death assessed by condensed chromatin in primary human neurons (**Fig. 5A**). However, these mutants also did not protect against Bax-mediated condensed chromatin (**Fig. 5A**) or TUNEL positive staining (**Fig. 5B**) compared to WT PrP's. Because all mutants lost their ability to inhibit the downstream Bax-mediated events, these results inferred that the mutants were also unable to inhibit caspase activation. The cytosolic level of GSS PrP mutants could not be assessed in these cells because of the very low transfection efficiency and the high endogenous level of PrP in the human primary neurons. Nevertheless, these results show the loss of anti-Bax function in the GSS-associated PrP mutants in human primary neurons.

## Discussion

In this manuscript, we examined the anti-Bax function of GSS-associated PrP mutants. WT PrP inhibits the first step of Bax activation in human primary neurons and in MCF-7 cells [18]. We previously have shown that eleven CJD-associated PrP mutants with a methionine at codon 129, partially or completely lost their anti-Bax function but that this loss of function could be rescued by co-expressing the CJD mutant PrP with WT CyPrP or its cognate mutant CyPrP [31]. We had also tested the GSS-associated A117V mutant and determined that this mutant retained its anti-Bax function in MCF-7 cells and in human neurons. This suggested that GSS-associated mutants could retain their anti-Bax function and that the retention of this protective function might be relevant to the longer duration of GSS diseases. However, none of the eight additional GSS PrP mutants studied here prevented Bax-mediated DNA condensation or DNA fragmentation in primary human neurons. Because endogenous PrP is highly expressed in these neuron cultures, the results indicate that the GSS-associated mutants, similar to the CJD-associated mutants, have a dominant effect over WT PrP with respect to the anti-Bax function. We also confirmed the loss of anti-Bax function in MCF-7 cells. These cells do not contain detectable levels of endogenous PrP under normal culture conditions, thus eliminating possible effects of endogenous PrP [14]. In the MCF-7 cells, P102L^M^, P105L^V^, Y145stop^M^, and Q212P^M^ could not inhibit Bax-mediated condensed chromatin. However, four of the GSS-associated mutants, F198S^V^, D202N^V^, P102L^V^ and Q217R^V^, prevented Bax-mediated condensed chromatin. However, except for the P102L^V^, these mutants did not inhibit caspase activation, a step that is upstream of chromatin condensation in the Bax-activated death pathway. The results indicated that these mutants simply delayed the cell death pathway because, in addition to the caspase activation in condensed chromatin positive cells, some transfected cells showed caspase activation in non-condensed chromatin positive cells. In contrast to human neurons, the MCF-7 cells lack the Casp3 gene and thus do not undergo DNA fragmentation although condensation of chromatin occurs through Casp6 and Casp7 activation [38], [39]. The difference in cell death pathways is likely the reason for the slight difference in the results between MCF-7 cells and human neurons. Therefore, we conclude from the results in human neurons and in MCF-7 cells that the anti-Bax function of PrP mutants is not associated with the long duration of the GSS prion disease.

We also show in this paper that the loss of GSS mutants in inhibiting Bax-mediated condensed chromatin results from the inability of these mutants to generate CyPrP in MCF-7 cells. As shown previously with the eleven CJD-associated PrP mutants [31], the level of CyPrP generated from the GSS-associated mutants was inversely proportional to the level of Bax-mediated condensed chromatin in MCF-7 cells. We observed severely depressed CyPrP levels in P102L^V/M^, P105L^V^, Y145stop^M^. The results with PrP Y145stop are consistent with others that show that PrP Y145stop has a short half-life and accumulates in the nucleus, ER, and Golgi but not significantly in the cytosol [44], [45]. We also observed weak levels of Y145stop^M^ mostly in the nuclear fraction. In those mutants that did generate detectable CyPrP, most of the CyPrP likely resulted from retrotranslocation because CyPrP migrated at the expected size for deglycosylated PrP lacking the N-terminal or C-terminal signal peptides. Retention of the signal peptides would show a protein migrating at 28 to 30 kDa rather than at 23–25 kDa as we observe. Furthermore, western blots of CyPrP with anti-signal peptide antiserum gave only a weak signal compared to that obtained with the anti-PrP 3F4 antibody. The anti-Bax function could have been influenced also by the aggregation of CyPrP, a condition detected in GSS mutants expressed in other cell types [41], [42], [46]. However, we did not observe any detergent insoluble CyPrP indicating that aggregation was not significant in our experimental conditions. The inability of the Q212P mutant to prevent Bax-mediated condensed chromatin, despite generating significant levels of CyPrP (our results and [41]), was consistent with our previous studies showing that proline disruption of PrP's helix 3 structure inhibits PrP's anti-Bax function [30]. The consequences of defective retrotranslocation or loss of the pre-emptive quality control pathway in these mutants will need to be examined more closely. It is generally assumed that familial PrP mutations affect the folding and the stability of the mutants compared to WT PrP and this has been experimentally demonstrated for P102L, F198S, and Q217R PrP mutants *in vitro* and *in vivo* studies [42], [46]–[53]. We have shown that CJD mutants accumulate to normal levels on the cell surface while undergoing defective retrotranslocation [31]. Several signaling functions are attributed to cell surface PrP and could be affected by misfolded mutant PrP that have escaped the retrotranslocation or the pre-emptive quality control pathways [54]–[57].

Most importantly, we observed that the GSS-associated mutants that generated significant levels of CyPrP and prevented condensed chromatin could not prevent caspase activation. Therefore, the CyPrP generated from these GSS-associated PrP mutants did not have normal anti-Bax function. Indeed, the F198S^V^, Q217R^V^, and D202N^V^ mutants generated as much CyPrP as WT PrP^V^ and while this level was sufficient in preventing Bax-mediated caspase activation in WT PrP, it was not in mutant PrPs. Others have shown that PrP Q217R^V^ escapes the ER quality control and accumulates as an abnormally folded form in the post-Golgi compartment of neuroblastoma cells [42]. Consistent with our observations, a portion of PrP Q217R^V^ accumulates in the cytosol [43]. Because we have previously demonstrated that CyPrP is the main form of PrP with the anti-Bax function [26], we conclude that the mutant CyPrP generated from these three mutants are less efficient at inhibiting the Bax activation pathway than the WT PrP. Previously, we have shown that the helix 3 is necessary and sufficient to inhibit Bax-mediated cell death [30]. The F198S^V^, Q217R^V^, and D202N^V^ location in helix 3 is consistent with the observed loss of anti-Bax function in these mutants, despite their ability to generate CyPrP. Therefore, the loss of anti-Bax function in PrP mutants can occur through two mechanisms: low levels of CyPrP through decreased retrotranslocation or pre-emptive quality control and loss in anti-Bax activity for those mutants that can generate CyPrP.

We found it interesting to observe significant amounts of PrP in the nuclear fraction from Wt PrP and most mutant PrP-transfected cells. We can exclude the possibility of contaminating endoplasmic reticulum membranes in the nuclear fraction since BiP is not detected in the nuclear proteins. Possibly, nuclear PrP was translocated from the cytosol to the nucleus. Others have reported fluorescent-tagged PrP and C-terminally truncated forms of PrP in the nuclear and perinuclear compartments [44], [45], [58]–[60]. Nuclear localization was also observed for CyPrP generated by alternative initiation of translation [61]. Two independent nuclear localization signals (NLS) in full length PrP were proposed [58] but CyPrP translocates to the nucleus independently of these two NLS [62]. The nuclear PrP possibly occurs only under stress conditions [58]. Interestingly, PrP interacts with DNA, RNA [63], [64], and chromatin [62], [65] and may be involved in a yet unidentified function in the nuclear compartment. Once this function is determined, it will be interesting to assess the effect of nuclear mutant PrP. However, the level of nuclear PrP did not correlate with the level of condensed chromatin and thus is unlikely to be involved in the anti-Bax function.

In conclusion, we have excluded the possibility that the retention of PrP's anti-Bax function in GSS-associated mutants could be responsible for the longer duration of GSS disease relative to CJD. However, the fact that nineteen different single point mutations of the prion protein associated with CJD, GSS and FFI significantly inhibit PrP's anti-Bax function suggests that both trafficking and structural problems arise with mutations in the PrP.
